# Hamartoma of the breast in two patients: A case report

**DOI:** 10.3892/ol.2013.1366

**Published:** 2013-05-29

**Authors:** FATMA CAVIDE SONMEZ, ZUHAL GUCIN, PELIN YILDIZ, ZEYNEP TOSUNER

**Affiliations:** Department of Pathology, Faculty of Medicine, Bezmialem Vakif University, Istanbul 34093, Turkey

**Keywords:** hamartoma, breast, adenolipoma

## Abstract

Breast hamartomas are rare, benign, tumor-like nodules composed of glandular, adipose and fibrous tissue. The hamartoma was first described in 1971 as a lipofibroadenoma, fibroadenolipoma or adenolipoma, based on the predominant component of the breast tissue. Clinical findings resemble fibroadenoma and if there is a palpable mass, the patients may receive an immediate diagnosis. Ductal hyperplasia, apocrine metaplasia, calcification and adenosis may occur within the hamartoma, with rarer instances of lobular or ductal intraepithelial neoplasms. Although hamartoma is usually benign, a malignant transformation is possible. An excision and histological examination is necessary for the differential diagnosis and also for any epithelial lesions of the hamartoma. Simple excision is enough for treatment if there is no coincidental epithelial malignant lesion. The patients in the present study were treated by simple excision as there were no proliferative changes in the lesions. No recurrence or other problems were detected in the 18-month follow-up. The current study presents two cases of breast hamartoma that were diagnosed as an adenolipoma and a fibroadenolipoma, and then describes the macroscopic and microscopic observations of these lesions.

## Introduction

Hamartomas of the breast are rare, benign lesions (1). These growths are also referred to as lipofibroadenomas, fibroadenolipomas or adenolipomas, based on their predominant components (1–3). Hamartomas are composed of glandular, adipose and fibrous tissue (3) often in abnormal proportions (as malformations) (4). Mammographic examination usually describe these lesions as a circumscribed, non-homogeneous tumor; however, the sonographic apperance may vary (2). The current study presents two cases of breast hamartoma. Written informed consent was obtained from the patients.

## Case reports

### Case 1

**Figure 1.**
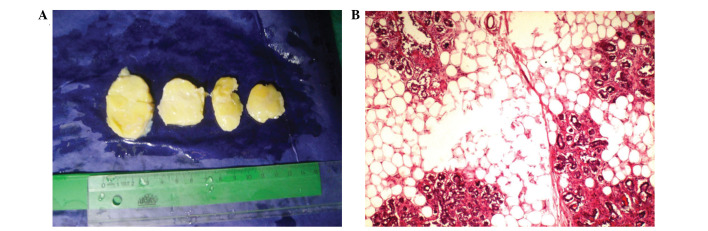
Gross and histopathological observations of adenolipoma. (A) Specimen reveals a well-defined, encapsulated, lobulated, yellow-colored mass resembling lipoma. The cut surface has small greyish-white areas. (B) Hematoxylin and eosin stained tissue from the lesion reveals benign lobulary aggregates in mature fatty tissue (magnification, ×40).

A 46-year-old premenopausal female was admitted to Karaman State Hospital, Karaman, Turkey with a lump in the left breast that had been detected by autodiagnosis. A physical examination revealed a round, mobile, regular mass in the upper outer quadrant of the breast. There was no axillar lymphadenopathy. Mammography identified a well-circumscribed mass in the left breast that was ∼8 cm in diameter and consistent with a lipoma. An ultrasonographic examination revealed that the breast mass was composed of isoechoic fat tissue, consistent with lipoma, and also revealed an intramammarian lymph node. The patient was treated by simple excision. Macroscopically, the tumor was a well-defined oval that was encapsulated, yellow in color and soft, measuring 5.2×5×2.5 cm in size. The cut surface was lobulated and yellow, with small greyish-white areas (Fig. 1A). The gross apperance of the tumor resembled that of a lipoma. Microscopic observations were also characteristic of a lipoma; the tumor was surrounded by a fibrous pseudocapsule and consisted of mature fat and islands of structurally normal glandular tissue with lobular arrangement (Fig. 1B). A fibrous stroma was found around the glandular tissue, however, in specific areas, lobular aggregates had direct contact with the fat cells without interference by the fibrous tissue. No proliferative changes in lobules and ducts were detected within the lesion. This case was consequently diagnosed as an adenolipoma.

### Case 2

**Figure 2.**
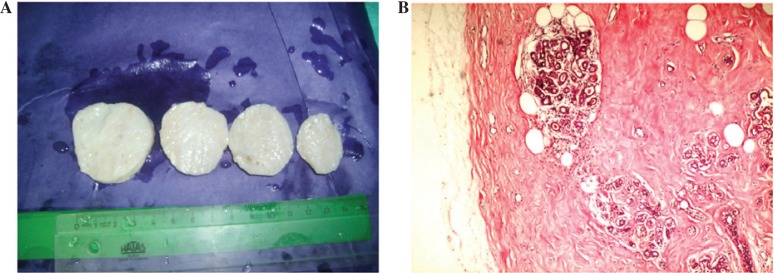
Gross and microscopic appearence of fibroadenolipoma. (A) Lesion is a well-circumscribed, encapsulated, greyish-white-colored mass resembling fibroadenoma. Note the small cystic spaces on the cut surface. (B) Hematoxylin and eosin stained mammary glandular tissue with mature adipocytes in hyalinized fibrous tissue (magnification, ×40).

A 41-year-old female was admitted to the same hospital with a palpable right breast mass. A physical examination revealed a round mass of ∼7 cm in diameter. No palpable lymph nodes or other masses of the contralateral breast were detected. The ultrasonography of the lesion revealed a solid, heterogeneous echogenic mass with smooth margins, measuring 5×2 cm. A excisional biopsy was performed on the right breast, and a 6.7×4.6×4.5-cm, greyish-white colored, oval-shaped, encapsulated mass was found upon gross examination (Fig. 2A). In addition, small cystic spaces were detected on the cut surface. Histopathologically, the tumor consisted of mammary glandular tissue in hyalinized fibrous stroma, interspersed with islands of mature fatty tissue (Fig. 2B). Cystic ducts with apocrine metaplasia were evident in specific areas as fibrocystic changes. All lobules were structurally normal. The lesion was well-defined and had a pseudocapsule of compressed adjacent breast tissue. This case was consequently diagnosed as a fibroadenolipoma.

## Discussion

Hamartomas of the breast are uncommon benign tumor-like nodules, also known as fibroadenolipoma, lipofibroadenoma or adenolipomas (3). The reported incidence of breast hamartomas is 0.7% of all benign breast tumors in females (5). Hamartomas were first described in 1971 by Arrigoni *et al* in a study of 10 patients whose breast tumors clinically and grossly resembled fibroadenomas (6). The majority of these lesions occur in females >35 years old. At clinical examination, hamartomas are usually occult, but they may manifest as large, mobile, soft to firm masses (7). Tumors as large as 17 cm have been reported (8). Breast hamartomas have become more frequently diagnosed due to the increased use of mammography, but they may be mistaken for neoplasms (9). During mammography scans, hamartomas are identified as typically well-circumscribed, round to oval masses containing fat and soft tissue densities with a thin, radiopaque pseudocapsule (7). The sonographic appearance of breast hamartoma has been reported to be variable and non-specific (8,9). Upon gross examination, hamartomas are typically well-demarcated, occasionally lobulated lesions with smooth contours and an often rubbery greyish-white to yellow cut surface, resembling a fibroadenoma or lipoma (3,4). The two common variants of breast hamartoma are adenolipoma and chondrolipoma (8). Adenohibernoma and myoid hamartoma are rare variants of hamartoma (4,10). Upon gross examination, adenolipomas are soft, circumscribed, occasionally lobulated masses, bordered by a thin fibrous pseudocapsule. The cut surface reveals a variegated pattern of fat and fibrous breast parenchyma. Lesions with abundant fat resemble lipomas (8).

Upon microscopic examination, Arrigoni *et al* identified ‘mammary glanduler tissue with a prominent lobuler arrangement, fibrous stroma and fat in varible proportions’ (6). Pathological observations in previous studies are varied and include circumscribed fibrocystic disease, adenolipoma, fibroadenoma with fat and fibroadenoma with lobules, as reported by Jones *et al*. In the same case report, heterologous elements were identified as cartilage and smooth muscle (11). This circumscribed mass of breast tissue may reveal fibrocystic and atrophic changes; pseudoangiomatous hyperplasia is frequently observed (10). The lesion generates the impression of a ‘breast within a breast’ (4,10). Usual ductal hyperplasia, apocrine metaplasia, calcification, stromal giant cells and adenosis may be associated with hamartoma (4). Lobular intraepithelial neoplasms and ductal intraepithelial neoplasms have also been reported to occur within the hamartoma in rare cases (4,12) Although hamartomas are usually benign, malignant transformation is possible (2,5).

Adenolipomas are composed of mature fat and mammary parenchyma with pseudocapsules. Lobules and ducts are structurally normal, with little or no proliferative change. Adenoliomas differ from other mammary lesions that contain fat (4).

Surgical removal is the curative method for breast hamartomas (2,3). If there is a coincidental epithelial malignancy in the lesion, there is a potential for recurrence (3). There was no recurrence or other problems in the 18-month follow-up of the patients in the present study. Excision and histological examination is necessary for a differential diagnosis and also for any epithelial lesions of the hamartoma.
